# Asthma diagnosis and treatment – 1024. Prevalence of depression among asthma patients and effects of asthma control on severity of depression

**DOI:** 10.1186/1939-4551-6-S1-P23

**Published:** 2013-04-23

**Authors:** Poonam Kumar, Swapnendu Misra, Susmita Kundu, AG Ghoshal, Debabrata Majumdar

**Affiliations:** 1 Respiratory Diseases and Clinical Immunology Society, India; 2 Pulmonary Medicine, Kolkata, India; 3 Pulmonary Medicine, R G Kar Medical College, Kolkata, India; 4 National Allergy Asthma Bronchitis Institute,Kolkata, India; 5 Psychiatry, Kolkata, India

## Background

Asthma is a serious global health problem. Global prevalence of asthma ranges from 1% to 18% of population in different countries.

In India, prevalence of asthma is 3% of the population. Major depressive disorder is the most common mood disorder often found to be higher among people with chronic health conditions like asthma. Presence of depression may lead to increased severity of asthma making it an uncontrolled asthma.

Our objective was to see prevalence of depression among asthma patients and effect of asthma control on severity of depression.

## Methods

All patients who met the inclusion criteria evaluated with Goldberg’s The General Health Questionnaire (GHQ 28), Bengali Version of Beck Depression Inventory (BDI) and Holmes & Rahe’s Life Event Scale. Severity of asthma and level of asthma control determined as per GINA guidelines. Follow up was done after 3 months and patients were again evaluated with Goldberg’s The General Health Questionnaire (GHQ 28), Bengali Version of Beck Depression Inventory (BDI) and Holmes & Rahe’s Life Event Scale.

## Results

Among 100 patients 35 (35%) had normal BDI score, mild mood disturbance was found in 23 (23%), borderline clinical depression in 12 (12%), moderate depression in 20 (20%), severe depression in 9 (9%), extreme depression in 1 (1%) patients. Follow up done at 3 months showed 68 patients had controlled and 32 patients had partially controlled asthma. Follow up evaluation with BDI showed 37 (37%) patients had normal score. Mild mood disturbance was found in 24 (24%), borderline clinical depression in 16 (16%), moderate depression in 14 (14%), severe depression in 8 (8%), extreme depression in 1 (1%) patients.

There is no significant correlation between severity of asthma and severity of depression (Correlation coefficient 0.047, p value 0.322).

There is also no correlation of asthma control on severity of depression (Correlation coefficient -0.036, p value 0.362).

## Conclusions

Depression is highly prevalent among asthma patients. There is no significant correlation between severity of asthma and severity of depression. There is also no correlation of asthma control on severity of depression.

